# A systematic review of the health effects of lead exposure from electronic waste in children

**DOI:** 10.3389/fpubh.2023.1113561

**Published:** 2023-04-12

**Authors:** Belay Desye, Amensisa Hailu Tesfaye, Gete Berihun, Ayechew Ademas, Birhanu Sewunet

**Affiliations:** ^1^Department of Environmental Health, College of Medicine and Health Sciences, Wollo University, Dessie, Ethiopia; ^2^Department of Environmental and Occupational Health and Safety, Institute of Public Health, College of Medicine and Health Sciences, University of Gondar, Gondar, Ethiopia

**Keywords:** lead, heavy metals, children, health effects, electronic waste, exposure

## Abstract

**Introduction:**

Electronic waste (e-waste) is an emerging human and environmental problem. Lead (Pb) is one of the most dangerous chemicals for human health, and it is the most prevalent heavy metal pollutant in e-waste. Despite the rapid growth of e-waste globally and the health effects of Pb, there is little information regarding the effects of Pb exposure from e-waste on children. Therefore, the aim of this review was to provide concise information on the health effects of Pb exposure from e-waste on children.

**Methods:**

A comprehensive search of databases was undertaken using PubMed/MEDLINE, Cochrane Library, Science Direct, HINARI, African Journal Online (AJOL), and additional sources were searched up to November 25, 2022. Eligibility criteria were determined using Population, Exposure, Comparator, and Outcome (PECO). The guidelines for Preferred Reporting Items for Systematic Reviews and Meta-Analysis (PRISMA) were used during the article selection process. The protocol of this systematic review was registered in the International Prospective Register of Systematic Reviews (Registration ID: CRD42022377028). The Joanna Briggs Institute (JBI) quality appraisal checklist was used to assess the quality of the included studies.

**Results:**

From a total of 1,150 identified studies, 20 full-text studies were included in the systematic review. All most included studies were conducted in China recycling area for e-waste. The included studies were conducted with an exposed group versus a reference group. The majority of the included studies found that blood Pb levels were ≥5 μg/dl and that Pb exposures from e-waste were affecting children’s health, such as a decrease in serum cortisol levels, inhibition of hemoglobin synthesis, impact on neurobehavioral development, affect physical development, etc.

**Conclusion:**

Lead exposure had a significant impact on children’s health as a result of informal e-waste recycling. Therefore, formalizing the informal sector and raising public health awareness are important steps toward reducing Pb exposure from e-waste. Moreover, the concerned stakeholders, like national and international organizations, should work together to effectively manage e-waste.

## Introduction

Electronic waste or Waste Electronic and Electrical Equipment (WEEE) refers to used and end-of-life electronic and electrical products (1). As Puckett et al. (2) define e-waste as “a broad and growing range of electronic devices ranging from large household devices such as refrigerators, air conditioners, cell phones, and consumer electronics to computers that have been discarded by their users.” Electronic waste is classified as a hazardous substance that requires special disposal due to the presence of a full spectrum of essential metals. It contains more than 1,000 different substances like Pb, mercury, cadmium, and brominated flame retardants (BFRs) like poly-brominated diphenyl ethers (PBDEs) (3–5). Electronic waste handling is a very complex and difficult issue due to its composition. Mishandling, informal recycling, and inappropriate disposal of e-waste can cause a serious impact on the environment and human health (1, 6).

Lead is the major heavy metal pollutant in e-waste and is found in cathode-ray tubes, circuit boards, batteries, and solder (7). In addition, Pb exposure is also found in the recycling of e-waste and acid batteries and in industrial settings like mining and smelting. Pb exposure can also occur in non-industrial settings such as hospitals, schools, and homes (8). According to the World Health Organization (WHO), Pb is one of the ten hazardous chemicals of public health concern (9, 10). Its widespread application has caused significant public health effects and environmental contamination (10). Due to its persistent nature, Pb can remain in environmental components such as water, soil, and air for a long time (11–14). Through ingestion, inhalation, and skin contact, Pb can enter the human body (11, 15).

Children living in the area of informal e-waste recycling, such as acid leaching, burning, roasting, and dismantling, are among the most at risk of exposure to Pb (11, 15). Even if they are not directly involved in recycling, children who live in home-based family workshops and communities where such e-waste recycling occurs can be easily exposed (16). Due to their immature systems and the fact that they have more exposure routes, like the habit of putting their hands in their mouths, children can be more vulnerable to Pb exposure. Even at low levels of Pb, children absorb 4–5 times as much Pb as adults from a typical source and experience the effects of Pb poisoning earlier than adults (10, 17).

Children are vulnerable to irreversible neurological and behavioral effects due to Pb. Lead exposure in children can have serious health consequences. It is mainly affecting brain development, which can reduce intelligence quotient (IQ), reduce learning ability, shorten attention spans, and increase the risk of behavioral problems (8). Lead exposure also affects hematological, neurological, gastrointestinal, renal, and cardiovascular organ systems (18) and causes the health effects of anemia, immune toxicity, hypertension, and toxicity to the reproductive organs (8).

Many countries around the world have laws and regulations requiring them to dispose of e-waste. Because of the high cost of recycling and the lack of legislative enforcement and disposal sites, e-waste is transported internationally from high-income countries to low-income countries with low environmental standards. Electronic waste recycling and disposal are emerging in regions beyond Asia, in many countries (19). Despite the fact that the Basel Convention prohibits the export of hazardous wastes to low-income countries (4).

Due to the fast development and advancement of the global electronics sector and technology, e-waste has become the fastest and largest growing amount of waste in the world (20, 21). In 2019, it was estimated that 53.6 million metric tons (Mt) of e-waste were generated globally, an average of 7.3 kg/capital, with 83% not properly collected and recycled (22). This indicated that recycling activities do not correspond to the global growth of e-waste. This informal management system may cause significant damage to the most vulnerable groups, like children (1). Significant amounts of Pb substances may be released into the environment as a result of these informal practices, negatively impacting children’s health (13).

Around 800 million people worldwide (1 in every 3 children) have high blood Pb levels (8). In many parts of the world, Pb has caused extensive health problems and environmental contamination (23, 24). The WHO reported that about 1 million people die every year from Pb poisoning (8). According to the Institute for Health Metrics and Evaluation (IHME), there were over 0.9 × 10^6^ deaths and 21.7 × 10^6^ Disability-Adjusted Life Years (DALYs) globally due to Pb exposure reported in 2019 (25).

Earlier findings revealed that Pb exposure in the recycling area of e-waste affects the health conditions of children, such as an increase in child sensory integration difficulties (26), cardiovascular endothelial inflammation (14), a decrease in child olfactory memory (27), and inhibition of hemoglobin synthesis (28). Because of the rapid and fast growth of e-waste globally and the health effects of Pb, there is a need to provide comprehensive and updated information about Pb exposure from e-waste activities. According to a database search, no systematic review on the health effects of Pb exposure in children has been conducted. Such studies can help policymakers and future researchers by providing detailed information about the health effects of Pb from e-waste in children. As a result, the aim of this study was to review the health effects of Pb exposure from e-waste on children.

## Review methods

### Registration and protocol

For this systematic review, the PRISMA guidelines were followed (29) (Supplementary File S1). The protocol of this systematic review was registered in the International Prospective Register of Systematic Reviews (PROSPERO) (Registration ID: CRD42022377028).

### Search strategies

A comprehensive search of databases was undertaken using PubMed/MEDLINE, Cochrane Library, Science Direct, HINARI, and AJOL, which were searched up to November 25, 2022. For the PubMed/MEDLINE search, the Boolean operators “AND” and “OR” and the following key terms were used in combination. ((Health effects (MeSH Term) OR Health Consequence [All Fields] OR Health Outcome [All Fields]) AND (Electronic waste [All Fields] OR E-waste [All Fields] OR Waste electronic and electrical equipment [All Fields] OR WEEE [All Fields]) AND Children [All Fields]).

In addition to the electronic database search, grey literature was searched using direct Google Search and Google Scholar. Bibliographies from the included articles listed as references were also searched to obtain additional studies.

### Eligibility criteria

Inclusion Criteria: In this review, studies that fulfilled the following criteria were considered for inclusion.

- Population: Children.
- Exposure: Pb from e-waste sources
- Comparator: The reported reference group (non-exposed group) for each study.
- Outcomes: Articles reported the health effects of Pb exposure from e-waste in children (health outcomes of Pb from e-waste in children).
- Study Design: A cross-sectional study.
- Time frame. There was no restriction on the study date. All studies reported up to November 25, 2022, were considered.
- Language of published articles: Only full-text articles written in English were considered.
- Publication issue: Peer-reviewed journal articles published until November 25, 2022.

Exclusion criteria: Qualitative studies, letters to editors, systematic reviews, short communications, and commentaries were excluded. In addition, studies that were not fully accessible after three personal email contacts with the corresponding author and studies that did not indicate the health effects of Pb exposure from e-waste on children were excluded.

### Study selection

Two investigators (BD and AHT) screened eligible studies independently by their title, abstract, and full text using predetermined eligibility criteria. The screened studies were compiled together by two investigators (BD and AHT), and the disagreement between authors that arises during data abstraction and selection is solved based on evidence-based discussion and the involvement of a third person (GB).

### Data abstraction/extraction

**Figure 1:**
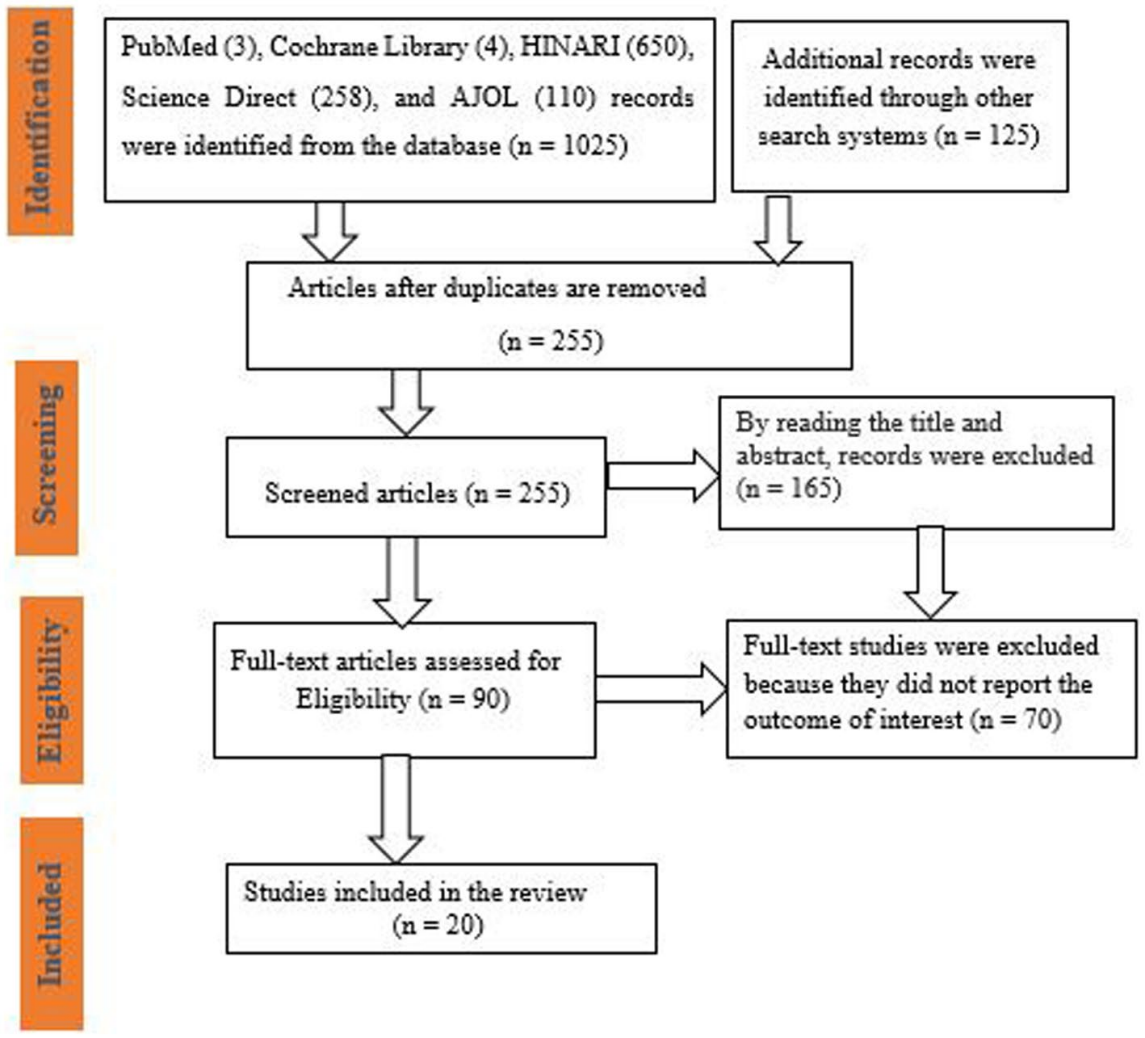
Flow diagram for the selection of studies for a systematic review of the health effects of Pb exposure from e-waste in children.

The data extraction format was included (name of the author and publication year, study country, study design, exposure setting, sample size, blood Pb levels, and health effects) (Table 1). Zotero reference manager software was used to collect and organize studies and for the removal of duplicate studies. The PRISMA flow diagram was used to summarize the selection process (Figure 1).

**Table 1:** Summary of 20 articles included in the review process of the health effects of Pb exposure from e-waste in children.

Author, publication year	Study country	Sample size	Age of participant	Blood lead levels	Health effects/ outcomes
Huo et al., 2019(30)	China, Guiyu and Haojiang	267 (exposed group, *n* = 132) and (reference group, *n* = 135)	2–7 years	Median 6.5 μg/dl for exposed groups and 4.4 μg/dl for exposed groups	Affect erythrocyte and stimulate cytokine secretion
Zheng et al., 2019 (14)	China, Guiyu and Haojiang	203 (exposed group, *n* = 105) and (reference group, *n* = 98)	3–7 years	Median 7.2 μg/dl for exposed group and 3.9 μg/dl for reference group	Exacerbated vascular endothelial inflammation
Wang et al., 2021 (28)	China, Guiyu and Haojiang	426 (exposed group, *n* = 222) and (reference group, *n* = 204)	3–6 years	Media 12.26 μg/dl for exposed group and 12.58 μg/dl for reference group	Inhibition of hemoglobin synthesis and may lead to anemia
Cai et al., 2019 (26)	China, Guiyu and Haojiang	574 (exposed group, *n* = 358) and (reference group, *n* = 216)	3–6 years	Median 4.88 μg/dl for exposed group and higher than the 3.47 μg/dl blood lead level for reference groups	Affect the child sensory integration and reduce in serum cortisol levels
Cao et al., 2018 (31)	China, Guiyu and Haojiang	118 (exposed group, *n* = 62) and (reference group, *n* = 56)	3–7 years	<10 μg/dl for exposed group	Higher percentages of peripheral CD4+ and CD8+ central memory T cells
Dai et al., 2017 (32)	China, Guiyu and Haojiang	484 (exposed, *n* = 332) and (reference group, *n* = 152)	2–6 years	Median 6.5 μg/dl for exposed group and 4.8 μg/dl for reference group	Higher blood and erythrocyte Pb levels of children adversely affected complement receptor expression in all children.
Zheng et al., 2008 (33)	China, Guiyu and Chendian	278 (exposed group, *n* = 154) and (reference group, *n* = 124)	<8 years	≥10 μg/dl for exposed groups	A serious threat to children’s health
Xu et al., 2018 (34)	China, Guiyu	118 (exposed group)	3–6 years	>5 μg/dl for exposed group	Oxidative DNA damage, potentially increasing the risk for mutations and cancer later in life
Yang et al., 2013 (35)	China, Guiyu	246 (exposed group)	3–8 years	Mean 7.3 μg/dl for exposed group	Affected children physical development and increased bone resorption of children
Zeng et al., 2018 (36)	China, Guiyu and Haojiang	466 (exposed group, *n* = 331) and (reference group, *n* = 135)	3–7 year	Median 5.64 μg/dl for exposed groups and 3.68 μg/dl for reference groups	Increased the problem of an amplified coagulation system through the activation of platelets
Zeng et al., 2019 (37)	China, Guiyu and Haojiang	470 (exposed group, *n* = 300) and (reference group, *n* = 170)	average 4.7 years	6.8 μg/dL for exposed group	Limits the growth and development of children
Zhang et al., 2017 (27)	China, Guiyu and Haojiang	118 (exposed group, *n* = 61) and (reference group, *n* = 57)	4–7 years	Mean 9.4 μg/dl for exposed group and 5.04 μg/dl for reference group	Affect the olfactory memory of children
Zhang et al., 2016 (38)	China, Guiyu and Haojiang	411 (exposed group, *n* = 285) and (reference group, *n* = 126)	3–7 years	6 μg/dl for exposed group and 3.92 μg/dl for reference group	Decrease the percentages of natural killer cells
Zeng et al., 2017 (12)	China, Guiyu and Haojiang	206 (exposed group, *n* = 100) and (reference group, *n* = 106)	5–7 years	Mean 5.53 μg/dl for exposed groups and 3.57 μg/dl for reference groups	Low levels of hemoglobin and affect lung function
Zhang et al., 2015 (39)	China, Guiyu	243 (exposed group)	3–7 years	7.9 μg/dl for exposed groups	Impact on neurobehavioral development
Lu et al., 2018 (40)	China, Guiyu and Haojiang	590 (exposed, *n* = 337) and (reference group, *n* = 253)	3–7 years	7.14 μg/dl for exposed groups and 3.9 μg/dl for reference groups	Abnormal measures of cardiovascular like vascular inflammation and lipid disorder
Liu et al., 2014 (41)	China, Guiyu	240 (exposed group)	3–7 years	7.35 μg/dl for exposed group	Behavioral abnormalities such as conduct problems and antisocial behavior
Liu et al., 2011 (42)	China, Guiyu and Chendian	303 (exposed group, *n* = 153) and (reference group, *n* = 150)	3–7 years	Median 13.2 μg/dl for exposed groups and 8.76 μg/dl for reference groups	Altering children temperament
Zhang et al., 2020 (43)	China, Guiyu and Haojiang	147 (exposed group, *n* = 73) and (reference group, *n* = 74)	3–7 years	Median 3.7 μg/dl for exposed groups and 2.3 μg/dl for reference group	Children may have dysregulated immune response and high inflammation risk
Daniell et al., 2015 (44)	Dong Mai, Vietnam	109	Not-specified	≥45 μg/dl for exposed groups	Affect the health of children

The study design of the include articles was a cross-sectional study and the study setting was conducted in the e-waste recycling areas.

### Assessment of methodological quality

The JBI quality appraisal tool for cross-sectional studies was used to assess the quality of included studies and the risk of bias in each study (45). Two reviewers (BD and AHT) independently assessed the quality of the included studies. The assessment tool contains eight criteria: (1) brief eligibility criteria; (2) description of the study participants and study area; (3) use of a valid and reliable method to measure the exposure; (4) standard criteria used for measurement of the variables; (5) identification of confounding factors; (6) development of strategies to control confounding factors; (7) use of a valid and reliable method to measure the outcome of interest; and (8) use of appropriate statistical analysis. It was evaluated using the JBI critical appraisal checklist options: Yes, No, Unclear, and Not Applicable. The risks for biases were classified as low (total score, 5–8) and high (total score, 0–4). The study scored 50% or higher on all quality-assessed items that were considered low-risk and included in this review (Supplementary File S2).

## Results and discussions

During the searching process, a total of 1,150 studies were identified. They were identified from PubMed, Cochrane library, HINARI, Science direct, AJOL, and additional records identified through other sources. From a total of 1,150 articles, 255 articles were remaining after removal of duplication. Of the remaining 255 articles, 165 were excluded after reviewing the titles and abstracts because they were not related. Moreover, 70 full articles were excluded for not meeting the inclusion and exclusion criteria. Lastly, 20 articles were included in this systematic review (Figure 1).

All most included studies (*n* = 19) were conducted in China, and (*n* = 1) was conducted in Vietnam. The included articles were conducted using a cross-sectional study design, and the studies were conducted in e-waste recycling areas. The included studies were conducted with an exposed group versus a reference group (non-exposed group). The majority of the included articles revealed that blood Pb levels were found to be ≥5 μg/dl and that Pb exposure from e-waste had an impact on children’s health, such as a decrease in serum cortisol levels, inhibition of hemoglobin synthesis, impact on neurobehavioral development, affect physical development, etc. (Table 1).

### Health effects of children’s from Pb exposure

The health effects of Pb exposure from e-waste on children have the features of multisystem and long-term effects (Table 1). Lead can have a significant impact on the health of children who live in an e-waste prone area. Children are more vulnerable to environmental exposures compared with adults due to more exposure routes (for example, placental absorption, breastfeeding, and finger sucking) and their immature systems and higher basal metabolic rates, which might make it difficult for them to handle and defecate some hazardous materials appropriately (46). Children also have a greater need for time to develop diseases compared to adults, which could be affected by hazardous chemicals in childhood and go through multiple developmental stages and years (47). Children are the most easily exposed group, and they need extra safety and protection because they are more vulnerable to environmental contaminants like Pb from e-waste. Pb exposure during childhood can have long-term and negative effects on an individual’s health (11, 15, 48).

The major sources or hotspots for e-waste recycling worldwide were in Guiyu, China (14, 26, 28, 30). In informal recycling of e-waste, activities like dismantling, chipping, and acid leaching were carried out for the recovery of metals. They have caused severe pollution that has caused extensive health problems for the residents and the local environment (49). Children who are growing and developing quickly are more vulnerable to the negative effects of Pb exposure from recycling areas of e-waste (11, 15). In China, many of the informal e-waste recycling activities were carried out, and the health of children in these informal recycling areas of e-waste was significantly affected by Pb, and their blood Pb levels were extremely high compared to other reference groups (non-exposed groups) (32, 36, 38, 50). Globally, China is one of the most serious polluters of the environment, with serious consequences for children’s health. Most local children were exposed to toxic chemicals such as Pb and other non-biodegradable organic pollutants due to informal recycling areas of e-waste (51).

The informal recycling of e-waste leads to the emission of hazardous substances like Pb and biodegradable compounds into nearby environmental areas like air, soil, water, etc., and in the long term it may contribute to air pollution. According to Rautela et al. (52), about 83% of e-waste worldwide is recycled informally. Inappropriate e-waste management and disposal at landfill sites have caused significant risks to the environment and subsequently affected health (2, 53). According to Heacock et al. (1), the majority of the toxic chemicals released by electronic products have accumulated in the environment and may have negative health effects. Lead is extensively and widely used in many electrical equipment and products, and it can come from either e-waste parts or the process of informal e-waste recycling. Consumption pattern, exposure time, dietary differences, and lifestyle are all factors that influence daily intake in the human body (54).

As the country with the largest production and recycling of e-waste, China has neglected the damage of informal e-waste recycling to children’s health and the environment for many years and is now paying a heavy price for it. More than 72.0, 61.0, and 86.5% of children in the e-waste exposed group had blood Pb concentrations greater than 5.0 μg/dl (13, 30, 36), respectively, compared to non-exposed groups (Table 1).

Lead exposure from e-waste has been linked to many toxicological outcomes in a variety of organ systems in children, including growth and development, hematological, cardiovascular, respiratory, and vascular inflammation (12, 35, 37, 39, 41, 42). Lead exposure also has negative health effects in children, like inhibition of hemoglobin synthesis (12, 28), lower percentages of natural killer cells (38), a decrease in child olfactory memory (27), and an increase in child sensory integration difficulties (26). Children with higher blood Pb levels (≥5 μg/dl) were more likely than those with low Pb levels to have a decrease in serum cortisol levels (26), oxidative DNA damage (34), affect both developmental conditions and promote bone resorption, and affect temperament conditions (42) (Table 1).

In the present study, in an e-waste exposed area, children with a higher blood lead level were more likely to have a lower serum cortisol level when compared with children with a lower blood lead level. This finding is consistent with studies conducted previously (26, 55, 56). This finding suggests that lead exposure can alter the Hypothalamic–Pituitary–Adrenal (HPA) axis function, leading to lower than expected cortisol release. The HPA axis compensatory mechanism may reach adaptation phase, resulting in lower cortisol levels and reduced reduction (57, 58).

The main strength of this study is that it identified the health effects of Pb exposure from e-waste in children at a global scale by searching the articles rigorously and following recommended procedures and protocols. This review also has its limitations. This review included articles written in English only.

## Conclusion and recommendations

This review comprehensively examines the adverse health effects of Pb exposure from e-waste on children. Electronic waste is the fastest growing waste stream, which can threaten children’s health and contaminate the environment. Electronic waste is not just a problem in one country; it is a worldwide issue. Due to additional routes of exposure, children are particularly vulnerable and sensitive to Pb. As China is the main informal recycler of e-waste, synthesizing the evidence of the adverse effects of Pb exposure from e-waste in children could provide a useful lesson for other countries, and e-waste issues are emerging globally. Due to inappropriate management and informal recycling of e-waste, Pb exposure significantly affects the health of children. The health outcomes of Pb exposure from e-waste must be a concern for the international community. Therefore, to reduce the problems of Pb exposure from e-waste, the concerned stakeholders, like national governments and international organizations, should work together to effectively manage e-waste in a sustainable way by formalizing informal sectors. It is also essential to increase public awareness about the effects and disposal systems of e-waste through regulatory frameworks. Moreover, eco-friendly technology is needed for proper management of e-waste.

## Data availability statement

The raw data supporting the conclusions of this article will be made available by the authors, without undue reservation.

## Author contributions

BD was involved in the conceptual development, data abstraction and selection, reviewing of articles, and report writing. AHT, GB, AA, and BS were involved in guiding the work and manuscript writing. All the authors read and approved the manuscript.

## Conflict of interest

The authors declare that the research was conducted in the absence of any commercial or financial relationships that could be construed as a potential conflict of interest.

## Publisher’s note

All claims expressed in this article are solely those of the authors and do not necessarily represent those of their affiliated organizations, or those of the publisher, the editors and the reviewers. Any product that may be evaluated in this article, or claim that may be made by its manufacturer, is not guaranteed or endorsed by the publisher.
