# Supplementary material for: A systematic review of the health effects of lead exposure from electronic waste in children
Source: Front Public Health. 2023 Apr 12;11:1113561. doi: 10.3389/fpubh.2023.1113561 (PMC10130399; doi:10.3389/fpubh.2023.1113561)
Supplement: Supplementary file 1 [file Data_Sheet_1.PDF]

Results of JBI Quality Assessment for cross-sectional study

Studies	Clear eligibility criteria	Description of study subject and study setting	Valid and reliable method to measure the exposure	Standard criteria used for measurement of the condition	Identification of confounding factors	Develop of strategies to deal with confounding factors	Valid and reliable method to measured outcomes	Appropriate statistical analysis	Total score out of 8	Level of bias
Huo et al., 2019	Unclear	Yes	Yes	Yes	N/A	Yes	Yes	Yes	7	Low
Wang et al., 2021	Yes	Unclear	Yes	Yes	N/A	Yes	Yes	Yes	7	Low
Cao et al., 2018	Yes	No	Yes	Yes	N/A	No	Yes	Yes	6	Low
Dai et al., 2017	Unclear	No	Yes	Yes	N/A	No	Yes	Yes	5	Low
Cai et al., 2019	Unclear	No	Yes	Yes	N/A	No	Yes	Yes	5	Low
Liu et al., 2011	Unclear	No	Yes	Yes	N/A	No	Yes	Yes	5	Low
Lu et al., 2018	Unclear	No	Yes	Yes	N/A	No	Yes	Yes	5	Low
Liu et al., 2014	Unclear	No	Yes	Yes	N/A	No	Yes	Yes	5	Low
Huo et al., 2007	Unclear	Unclear	Unclear	Yes	N/A	No	No	Yes	3	High
Huo et al., 2014	Unclear	Unclear	No	No	N/A	No	No	Yes	2	High
Xu et al., 2018	Unclear	No	Yes	Yes	N/A	Yes	Yes	Yes	6	Low
Zeng et al., 2017	Unclear	Unclear	Yes	Yes	N/A	Yes	Yes	Yes	6	Low
Yang et al., 2013	Unclear	Unclear	Yes	Yes	N/A	Yes	Yes	Yes	6	Low
Zeng et al.,2018	Unclear	Unclear	Yes	Yes	N/A	Yes	Yes	Yes	6	Low
Zheng et al., 2019	Unclear	Unclear	Yes	Yes	N/A	Yes	Yes	Yes	6	Low
Zhang et al., 2017	No	Unclear	Yes	Yes	N/A	Yes	Yes	Yes	6	Low

Zheng et al., 2008	No	Unclear	Yes	Yes	N/A	Yes	Yes	Yes	6	Low
Zeng et al., 2019	Unclear	Unclear	Yes	Yes	N/A	Yes	Yes	Yes	6	Low
Zhang et al., 2016	Unclear	Unclear	Yes	Yes	N/A	Yes	Yes	Yes	6	Low
Zhang et al., 2015	Unclear	Unclear	Yes	Yes	N/A	Yes	Yes	Yes	6	Low
Zhang et al., 2020	Unclear	No	Yes	Yes	N/A	Yes	Yes	Yes	6	Low
Daniell et al., 2015	Unclear	Unclear	Yes	Yes	N/A	Yes	Yes	Yes	6	Low
